# ChIP-Seq reveals that QsMYB1 directly targets genes involved in lignin and suberin biosynthesis pathways in cork oak (*Quercus suber*)

**DOI:** 10.1186/s12870-018-1403-5

**Published:** 2018-09-17

**Authors:** Tiago Capote, Pedro Barbosa, Ana Usié, António Marcos Ramos, Vera Inácio, Ricardo Ordás, Sónia Gonçalves, Leonor Morais-Cecílio

**Affiliations:** 1 Centro de Biotecnologia Agrícola e Agro-alimentar do Alentejo (CEBAL) / Instituto Politécnico de Beja (IPBeja), Beja, Portugal; 2 Instituto de Ciências Agrárias e Ambientais Mediterrânicas (ICAAM), Universidade de Évora, Évora, Portugal; 3 Linking Landscape, Environment, Agriculture and Food (LEAF) Instituto Superior de Agronomia, University of Lisbon, Lisboa, Portugal; 4 Departamento BOS, Escuela Politécnica de Mieres, Oviedo University, Oviedo, Spain; 5 Present Address: Wellcome Sanger Institute, Wellcome Genome Campus, Hinxton, Cambridge, CB101SA UK

**Keywords:** Cork oak, Chromatin-immunoprecipitation, Transcription factor, R2R3-MYB, ABCG transporters, Lipid-protein transporters

## Abstract

**Background:**

Gene activity is largely controlled by transcriptional regulation through the action of transcription factors and other regulators. QsMYB1 is a member of the R2R3-MYB transcription factor family related to secondary growth, and in particular, with the cork development process. In order to identify the putative gene targets of QsMYB1 across the cork oak genome we developed a ChIP-Seq strategy.

**Results:**

Results provide direct evidence that QsMY1B targets genes encoding for enzymes involved in the lignin and suberin pathways as well as gene encoding for ABCG transporters and LTPs implicated in the transport of monomeric suberin units across the cellular membrane. These results highlight the role of QsMYB1 as a regulator of lignin and suberin biosynthesis, transport and assembly.

**Conclusion:**

To our knowledge, this work constitutes the first ChIP-Seq experiment performed in cork oak, a non-model plant species with a long-life cycle, and these results will contribute to deepen the knowledge about the molecular mechanisms of cork formation and differentiation.

**Electronic supplementary material:**

The online version of this article (10.1186/s12870-018-1403-5) contains supplementary material, which is available to authorized users.

## Background

Regulation of gene activity at the transcriptional level is the most common form of gene control. Regulation of transcription generally occurs via changes in the amounts and activities of transcription factors (TFs), which modulate the transcription of specific genes either activating or repressing the rate of transcription [1]. TFs interact and function in a combinatorial manner forming homo and heterodimers, assembling on control elements of DNA and recruiting cofactors [2, 3]. Cofactors in turn recruit DNA and histone modifying enzymes modulating the chromatin configuration state [1]. TFs also function in networks in which a protein may regulate the expression of another to control directly or indirectly the expression of a particular gene or group of genes in a temporal and spatial fashion manner, allowing the unique expression of each gene in different cell types and during development [1].

A distinct characteristic of TFs is that they present a specific DNA-binding domain, which confers them the ability to bind to specific DNA regions and controlling target genes. Chromatin immunoprecipitation (ChIP) followed by high-throughput DNA sequencing (ChIP-Seq) is a method widely used to identify the binding sites of a target protein across a genome. In a ChIP assay, a TF, cofactor, or other chromatin protein is enriched by immunoprecipitation from cross-linked cells along with its associated DNA [4]. The resulted DNA is then sequenced and mapped against the species genome in order to identify the binding sites of protein of interest. This allows the subsequent identification of their gene targets, unravelling potential regulatory networks [4].

Plant TFs are characterized by a larger number of genes and by the diversity of families. In *Arabidopsis* there are around 2000 TFs genes belonging to approximately 30 different TF families [5]. Most plant TFs as APETALA2/Ethylene Responsive element binding factor (AP2/ERF), NAC, MADS box, basic helix-loop-helix (bHLH), basic leucine zipper (bZIP) and myeloblastosis (MYB) form large domain families playing important roles in the control of plant growth and development [1]. The MYB family constitute the most abundant group of TFs in plants. In *Arabidopsis,* 198 MYB TFs were identified, of which 126 belong to the R2R3-MYB subfamily [6]. The evolution of R2R3-MYBs in plants seems to be related with a specific expansion of the subfamily giving rise to species-specific gene subgroups in certain species [7]. This expansion is mainly attributed to whole genome and segmental duplication as gene sequence and phylogenetic tree analyses confirm [7]. They have been classified into 28 subgroups according to the conserved amino acid sequence motifs present in C-terminal MYB domain [3, 8].R2R3-MYB TFs are characterized by their role in a variety of plant-specific processes, such as cell shape and morphogenesis, cellular proliferation and differentiation, hormone response, abiotic and biotic stress, and regulation of primary and secondary metabolism such as phenylpropanoid, lignin and suberin metabolism [9, 10]. However analysis of the promotor sequences in chinese pear demonstrated that transcriptional regulation of the MYB genes is variable among species [11].AtMYB41, for example, it is known to be involved although indirectly in the regulation of suberin biosynthesis, export, assembly and deposition in plants under stress conditions [12], however it not expressed in the poplar suberized tissue phellem [13]. It was recently reported a relation between AtMYB107 and AtMYB9 which synchronize the transcriptional induction of aliphatic and aromatic monomer biosynthesis as well as suberin transport and polymerization in seed outer integument layer [14]. Gou et al., [10] reported AtMYB107 as a positive regulator for seed coat suberin synthesis, highlighting the important role of MYB TFs in suberin synthesis regulation. In poplar at least 18 MYB transcription factors were reported as up-regulated in phellem with functions related with the suberin metabolic pathways [13]. Among these, Arabidopsis homologs of *AtMYB107*, *AtMYB9* and *AtMYB93* were found, which have regulatory functions in the suberization process [10, 14–16]. Also,homologs of *AtMYB96*, *AtMYB94, AtMYB106* and *AtMYB16* related with cuticle metabolism [17–19], homologs of *AtMYB3*, *AtMYB7*, *AtMYB63* linked to phenylpropanoid metabolism [20, 21], an homolog of root AtMYB36 which is related with root casparian band formation [22], and *AtMYB111* with a regulatory function in the flavonoid metabolism were identified [23].

Cork oak (*Quercus suber L.)* is an evergreen broadleaved tree species native to the Mediterranean basin. It is a valuable economic resource due to the sustainable exploitation of its thick bark, the cork [24, 25]. Cork or phellem is a tissue derived from the meristematic activity of the phellogen characterized by a layered deposition of suberized death cells [26]. The high content in suberin provides cork with unique insulator and elastic properties that translates into a large variety of industry applications. Despite the importance of cork, the knowledge on the molecular mechanisms on cork formation and development, are still poorly understood.

Almeida and co-workers (2013) [27] have characterized a R2R3-MYB gene previously identified as related with cork formation and differentiation [28], which was named QsMYB1. Authors showed that QsMYB1 is mainly active in organs and tissues with secondary growth resulting from the activity of phellogen [27]. Moreover, *QsMYB1* transcripts are more abundant in cork, a highly suberized tissue, than in wood, a lignified but non-suberized tissue. The authors have also found several *cis*-acting regulatory elements related to phenylpropanoid pathway in the *QsMYB1* promotor region [29]. QsMYB1 expression was also shown to be modulated in response to heat and drought stress, which points to a function in the regulatory network of cork oak response to abiotic stress [29]. These findings led the authors to hypothesize that QsMYB1 may be regulating one or more metabolic pathways involved in cork formation, namely the biosynthesis of lignin and suberin which constitute the major biopolymers present in cork.

The present work aims to validate these results, by developing a ChIP-Seq strategy to identify the QsMYB1 target genes. Results showed that QsMYB1 acts directly on genes of the lignin and suberin metabolic pathways, confirming the specific role of QsMYB1 in regulating cork formation and development, a specific biological process with great relevance in cork oak.

## Results

### QsMYB1::3xFLAG somatic embryo selection for ChIP-Seq

**Fig. 1:**
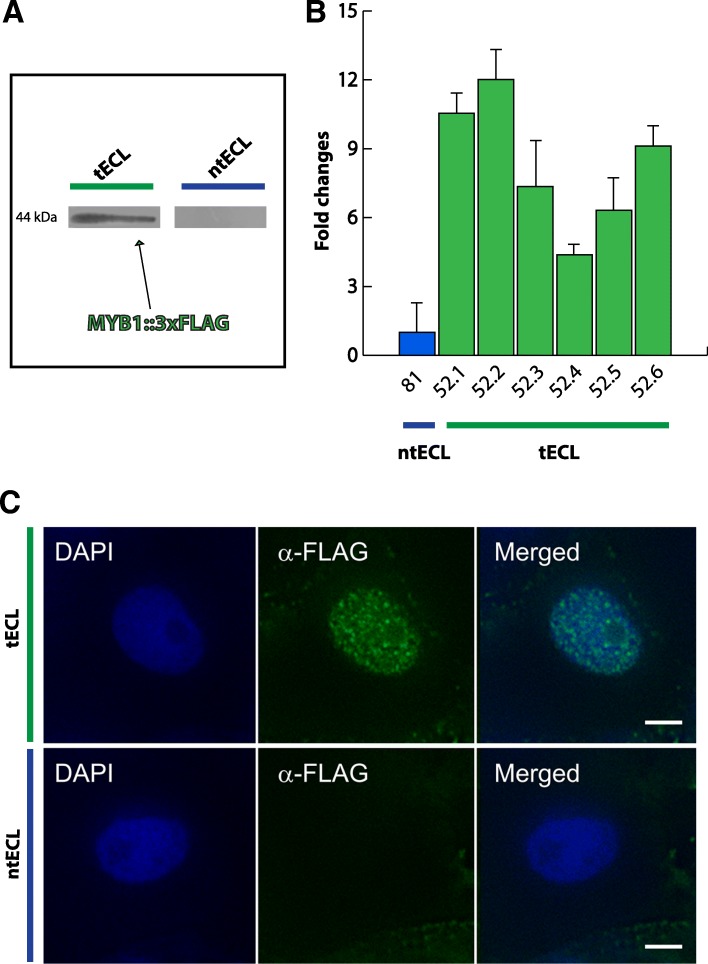
MYB1::3xFLAG transcripts and protein detection. **a** – Western Blot of MYB1::3xFLAG proteins in tECL. Total proteins from non-transformed ECL (ntECL) were used as negative control. **b** – MYB1::3xFLAG transcripts levels from six transformed embryogenic cell lines (tECL) clusters (52.1–52.6) quantified by RT-qPCR and normalised against non-transformed embryogenic cell lines (ntECL)(81). Mean and standard errors of three independent experiments are shown. **c** – MYB1::3xFLAG protein nuclear detection by fluorescence microscopy in tECL. Nucleus magnification is shown for tECL and ntECL. Bars = 5 μm

Stable modified cork oak somatic embryo cell lines overexpressing QsMYB1::triple FLAG fused (QsMYB1::3xFLAG) protein were produced using Ce1 line, previously generated (unpublished observation). Ce1 line, stable and with a high rate of secondary embryogenesis, was previously characterized for kanamycin resistance, allowing the determination of the somatic embryo natural resistance to grow under the presence of the antibiotic. Concentrations over 25 mg/ml of kanamycin showed to inhibit embryo proliferation setting kanamycin at this concentration as selective agent of putative transformants. After 30 days subcultures during 24 months, MYB1::3xFLAG transcripts were detected in several transformed embryo clusters by quantitative real time PCR (RT-qPCR). When comparing with the expression of *QsMYB1* in non-transformed embryos, MYB1::3xFLAG transcripts presented a 4–12 fold change in the level of expression (Fig. 1a). Furthermore, the specificity of the anti-FLAG antibody was validated by western blot (Fig. 1b) and the MYB1::3xFLAG protein was detected in the nucleus by fluorescence microscopy (Fig. 1c), confirming the nuclear location of the protein. Due to the lack of known target DNA of QsMYB1 and consequent impossibility to control the enrichment quality of ChIPed DNA, we selected embryo clusters with fold change expression of *QsMYB1::3xFLAG* higher than 6 (Fig. 1a) in order to have enough protein suitable to perform the ChIP-Seq assay.

### QsMYB1 target genes identified by ChIP-Seq

**Fig. 2:**
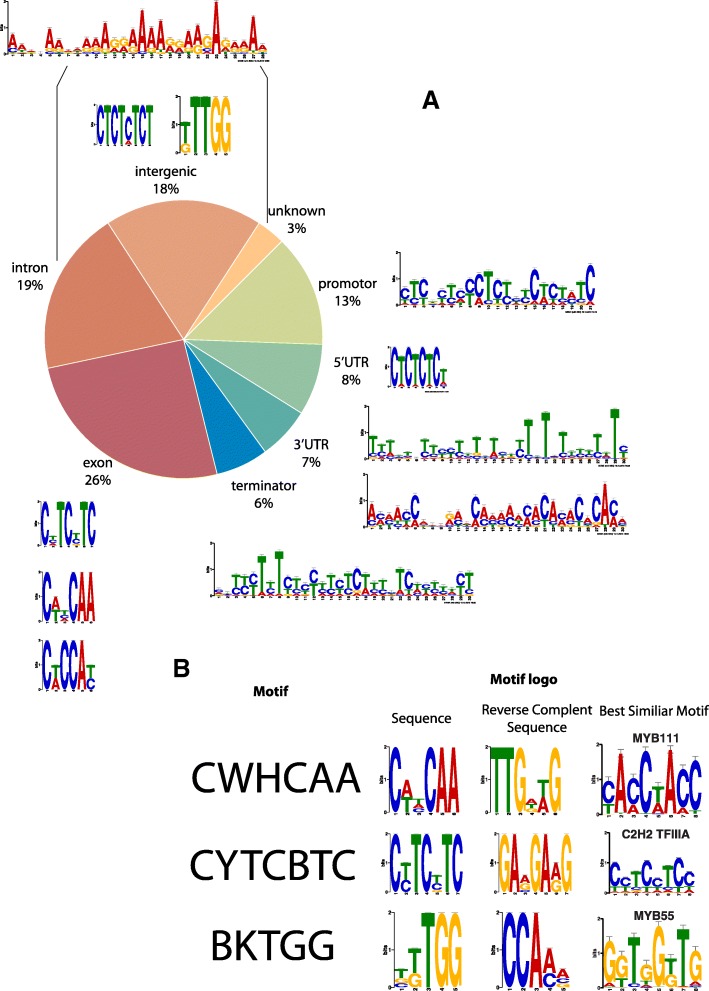
**a** Distribution of peaks by genic and intergenic regions and correspondent motifs of QsMYB1 preferentially binding. **b** Summary of motifs analysis of all peaks. The three more representative motifs of all peaks detected and the best similar motif known are represented

In order to identify the DNA targets of QsMYB1, ChIP-Seq was performed by immunoprecipitating QsMYB1 protein and the cross-linked DNA after chromatin fragmentation of ~ 300 bp (Additional file 1: A and B). DNA was purified and further sequenced with the Illumina HiSeq 4000 system producing ~ 70–90 million reads per sample of which ~ 123 million reads uniquely mapped to the cork oak draft genome (Table 1). One ChIP (anti-FLAG) and one mock (IgG) library were sequenced with one technical duplicate each. After reads, filtering peaks were called with the MACS2 and selected for downstream analysis according to the ENCODE ChIP-Seq guidelines (detailed description of data analysis in Methods section). A total of 18,165 putative binding sites were identified. It was firstly analysed whether the peaks in the ChIP experiment were distributed by genic regions. Based on these analyses, MYB1 binding sites are located in genic regions of 14,290 genes: 13.4% in promotor regions, 8.1% in 5′ untranslated regions (UTR), 19.2% in intron regions, 25.5% in exon regions, 6.5% in 3’ UTR and 6.0% in the terminator regions (Fig. 2a). Intergenic regions represent 18.4% of the total binding sites and 2.9% of the binding sites were not annotated (unknown) (Fig. 2a). The Peak-calling analysis identified several target genes reflecting the binding of QsMYB1 to specific DNA loci. Between these genes, QsMYB1 targets other TFs, genes encoding for enzymes related with the lipid metabolism and transport, as well as enzymes from the phenylpropanoid pathway. Amongst these genes, several are related with various aspects of cork formation, as suberin and lignin biosynthesis or transport and deposition of suberin monomeric units. In order to explore the regulatory functions of QsMYB1 in cork formation and differentiation we focused the analysis in these genes, however QsMYB1 potentially targets a vaster number of genes.

**Table 1:** Sequencing throughput and mapping results obtained for ChIP and mock samples

Sample	Raw reads	Pre-processed	UMR reads	% UMR
ChIP_L1	75,987,297	72,855,787	28,558,358	37.58%
ChIP_L5	66,520,992	63,594,856	24,887,132	37.41%
Mock_L1	93,072,373	89,005,882	37,335,088	40.11%
Mock_L5	80,189,676	76,512,503	32,053,662	39.97%

### Binding motif analysis reveals QsMYB1 *cis*-regulatory elements

To explore the MYB1 biding motifs environment, 1-kb flanking sequences around all peaks summit were analysed by the motif discovery tool MEME-ChIP using the DAP motifs [30] and PBM motifs [31] databases (Fig. 2b). Motifs CWHCAA (E-value = 3.5e-50), CYTCBTC (E-value = 8.8e-38) and BKTGG (E-value = 2.9e-30) were the most statistically relevant. The BKTGG motif is presented in 12,308 peaks (67.9%), while the CWHCAA and the CYTCBTC motifs were identified in 7926 (43.7%) and 2016 (11,1%) peak sequences, respectively. When the occurrence of motifs by binding locations is individually analysed, it is observed that some genic regions present more than one identity motif. Nevertheless, each binding feature has a similarity with the 3 global motifs identified (Fig. 2a).

### QsMYB1 directly targets other transcriptional elements

**Fig. 3:**
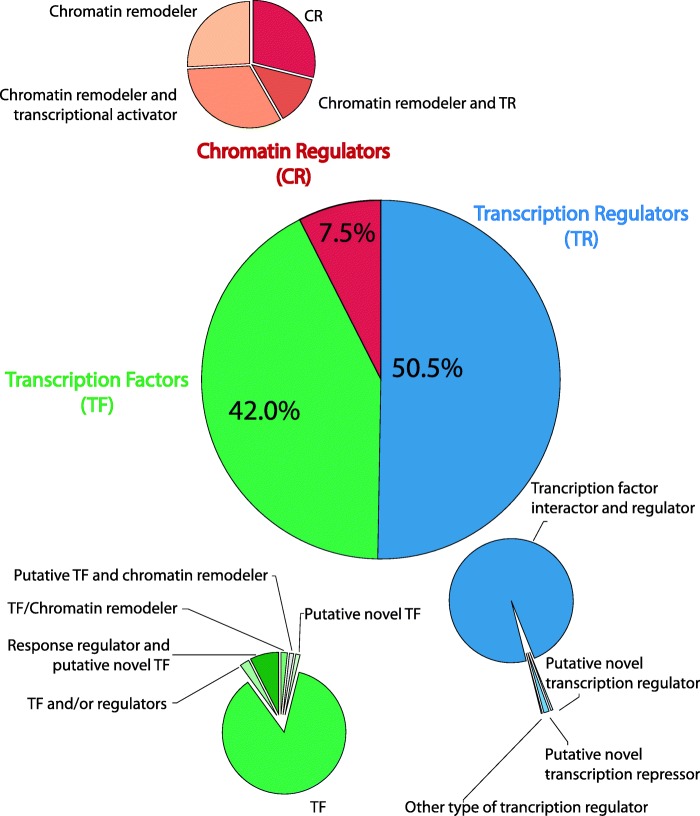
Types of transcription factors, transcription regulators and chromatin regulators targeted by QsMYB1

To detect genes regulated by QsMYB1 gene, the QsMYB1 DNA-targets putatively encoding for TFs, transcription regulators and chromatin regulators the genes associated with each peak were analysed with the PlantTFcat tool [32]. Results showed that 414 regulatory genes are targeted by QsMYB1: TF (42%), transcription regulators (50.5%) and chromatin regulators (7.5%) comprising different types of regulators families (Fig. 3). From these families the most representative putatively encodes for CCHC zinc finger proteins (CCHC) (192), C2H2-type zinc fingers TFs (C2H2) (43), WD40-like TFs (19), BED-type zinc finger proteins (BED-type(Zn)) (13), AP2/ERF TFs (10) and bHLH TFs (10) (Additional file 2).

### Enzyme encoding genes targeted by QsMYB1

**Fig. 4:**
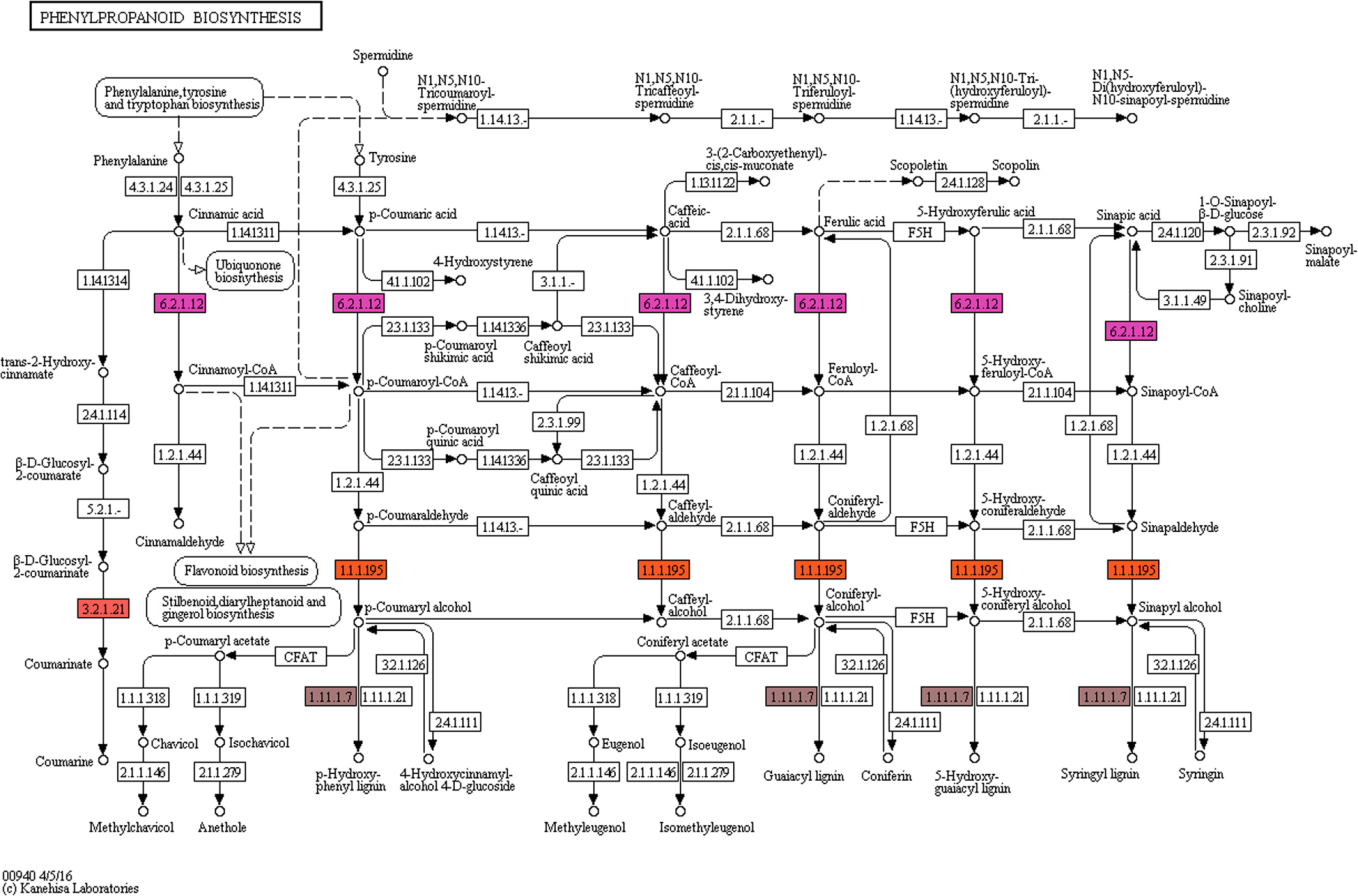
Enzymes target by QsMYB1 in the phenylpropanoid biosynthesis pathway (colored boxes). Numbers correspond to enzymes E.C. numbers

To identify the targets of QsMYB1 directly related with secondary growth and cork formation, all QsMYB1 putative target genes were submitted to the KEGG database giving focus to the ones that present a fold enrichment higher or equal to 3 (Additional file 3). With this approach, 16 genes encoding three distinct enzymes essential to the phenylpropanoid pathway (Fig. 4) were identified, namely 4-coumarate: CoA ligase (4CL - 6.2.1.12), cinnamyl alcohol dehydrogenase (CAD - 1.1.1.195) and class III plant peroxidase (PPO - 1.11.1.7). Also related with the phenylpropanoid metabolism, the results show that QsMYB1 putatively targets three distinct genes encoding for β-galoctosidase (β-GAL - 3.2.1.21).

**Fig. 5:**
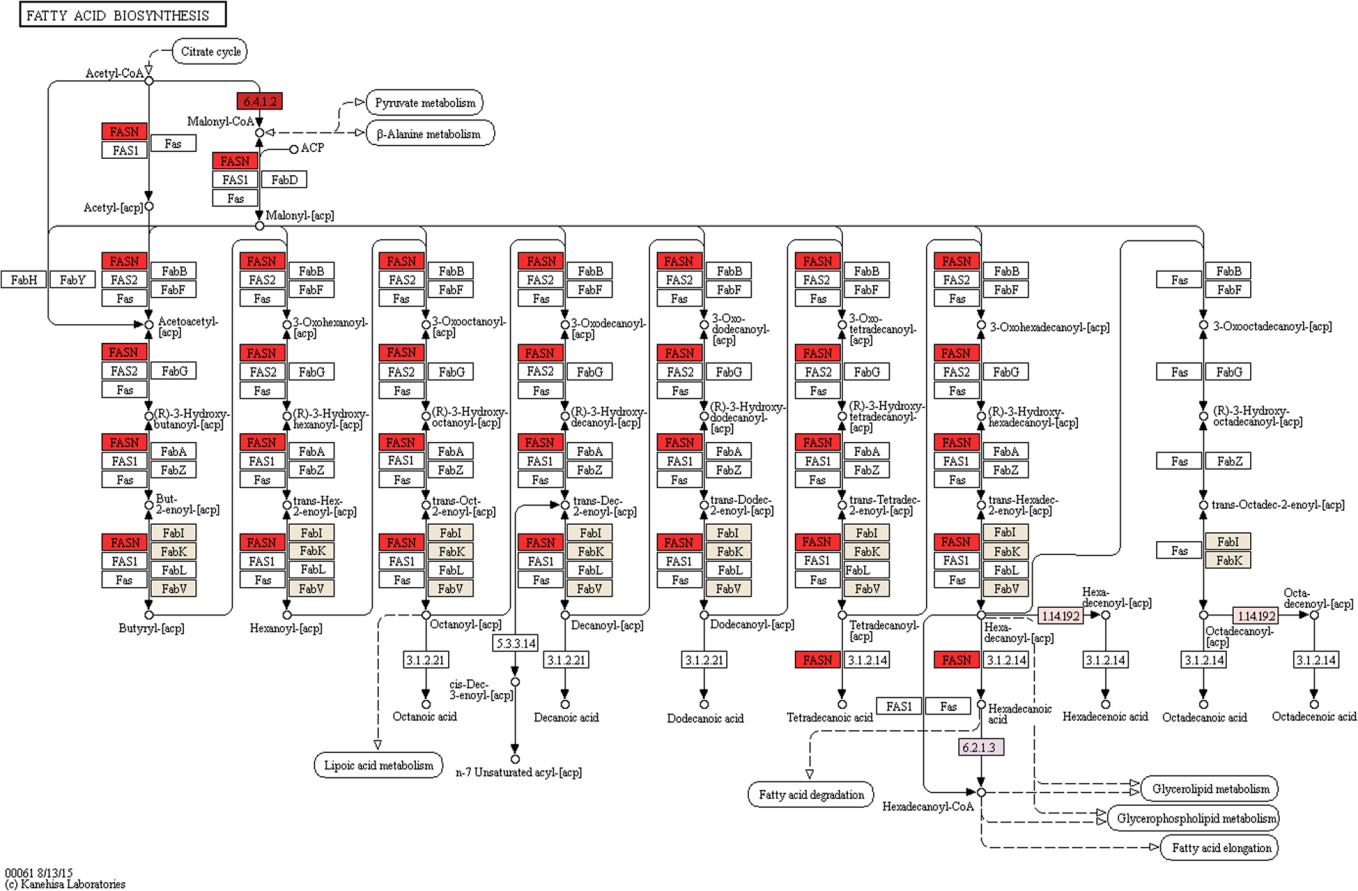
Enzymes target by QsMYB1 in the fatty acid biosynthesis pathway (colored boxes). Numbers correspond to enzymes E.C. numbers

**Fig. 6:**
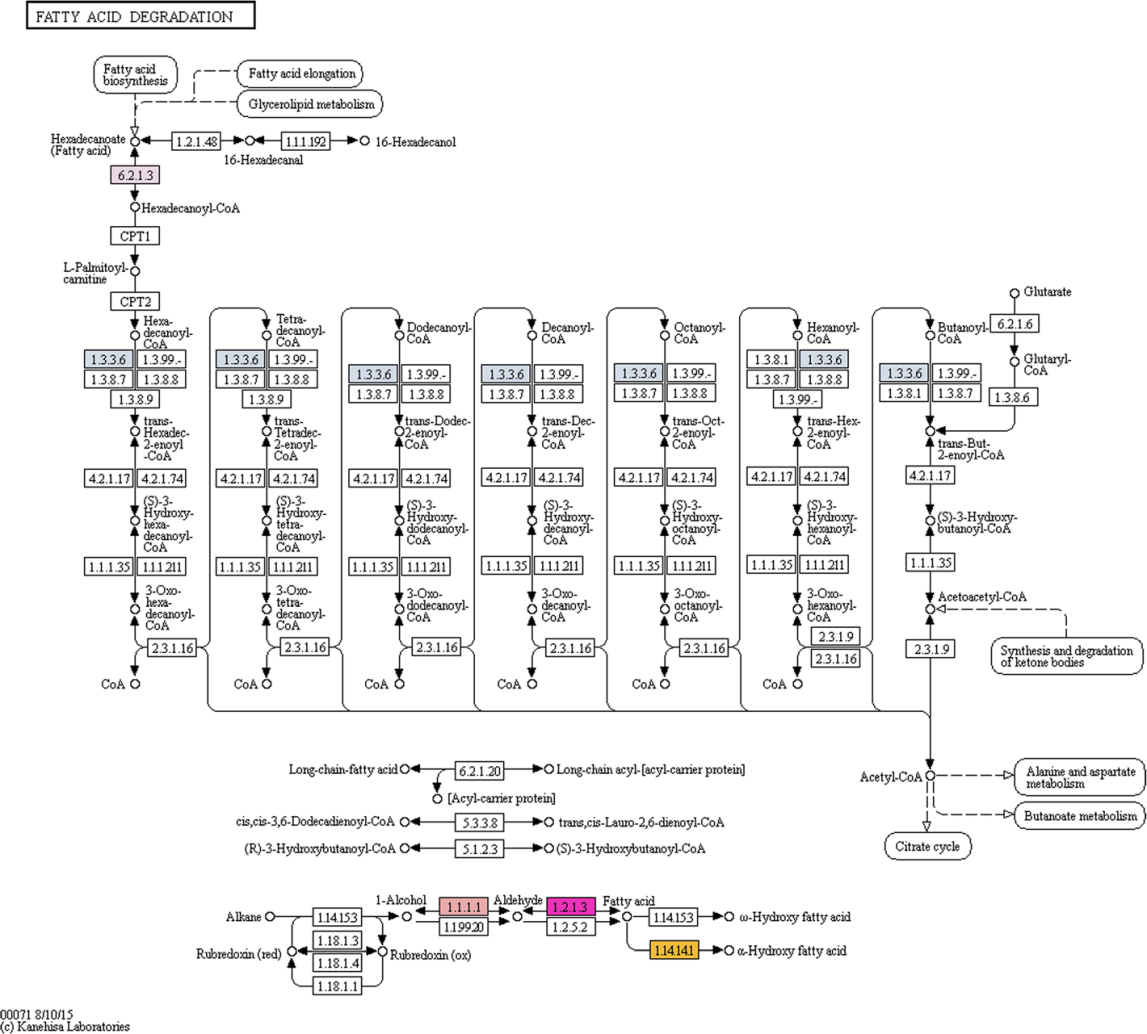
Enzymes target by QsMYB1 in the fatty acid degradation biosynthesis pathway (colored boxes). Numbers correspond to enzymes E.C. numbers

Diverse QsMYB1 target genes encoding for key enzymes on the metabolism of lipids were identified, namely enzymes related with fatty acid biosynthesis (Fig. 5) as acetyl-CoA carboxylase (ACC – 6.4.1.2), long-chain-fatty-acid-CoA ligase (LACS – 6.2.1.3) and members of fatty acid synthase complex: enoyl-[acyl-carrier-protein] reductase (NADH) (FABI – 1.3.1.9) and acyl-[acyl-carrier-protein] desaturase (ACP - 1.14.19.2). Moreover, we found two Arabidopsis homolog genes of β-ketoacyl-CoA synthase in our data. Related with fatty acid oxidation (Fig. 6), the results revealed two genes encoding for a putative acyl-CoA oxidase (ACX – 1.3.3.6) and two genes encoding for a aldehyde dehydrogenase (NAD+) (ALDH - 1.2.1.3). Also, two distinct target genes related the ω-hydroxylation of various fatty acids and with high homology for the Arabidopsis Cytochrome P450 (CYP) 86A8 (*AtCYP86A8*) and for the Arabidopsis CYP 96A1 (*AtCYP96A1*) were found.

**Fig. 7:**
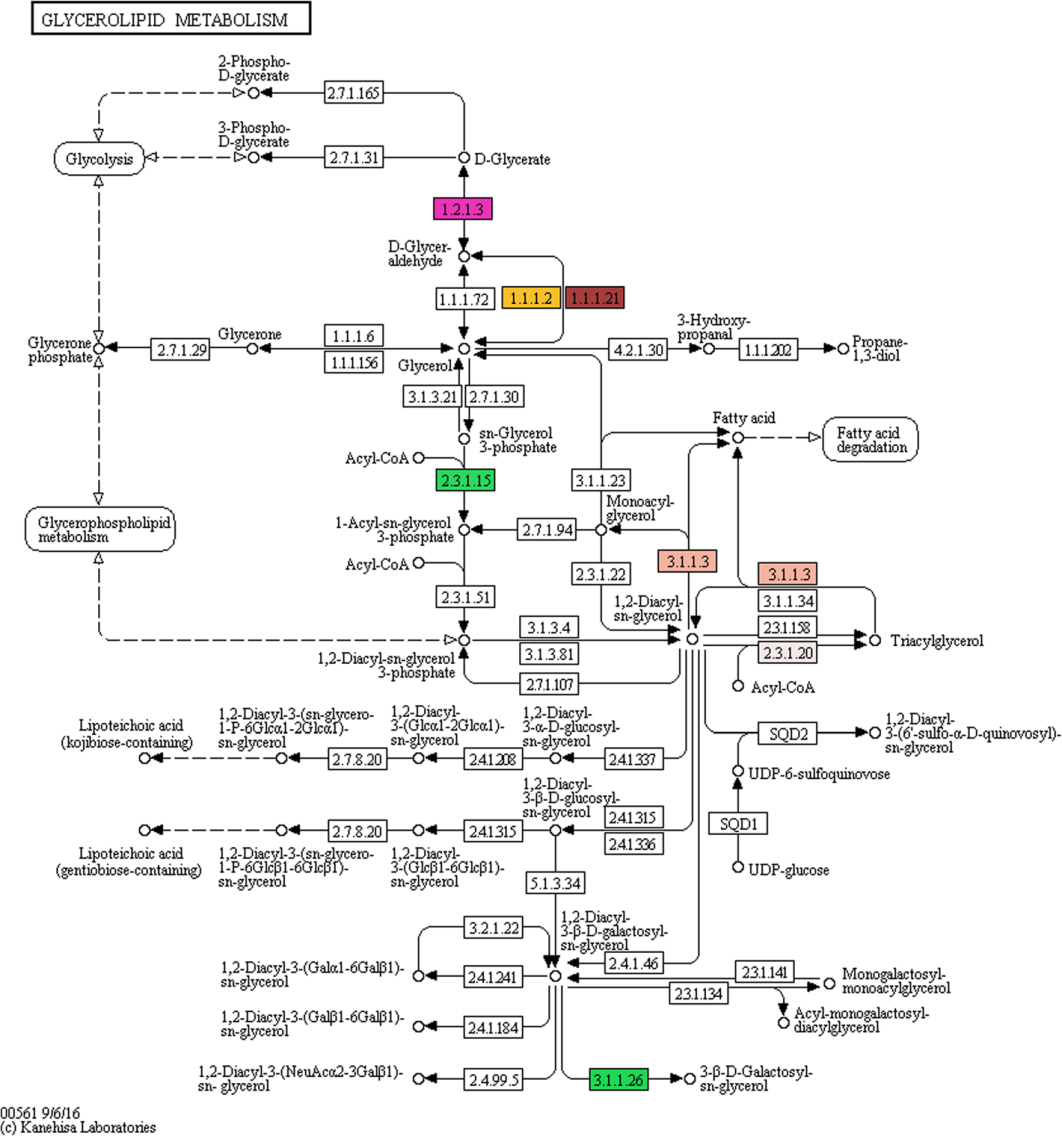
Enzymes target by QsMYB1 in the glycerolipid metabolism (colored boxes). Numbers correspond to enzymes E.C. numbers

The results additional show several genes encoding for enzymes involved in the glycerol metabolism (Fig. 7) as aldehyde dehydrogenase (NAD+) (ALDH - 1.2.1.3), NADP-dependent alcohol dehydrogenase (ADH – 1.2.1.1), NADPH-dependent aldehyde reductase (ADR – 1.1.1.21), glycerol 3-phosphate acyltransferase (GPAT – 2.3.1.15), 1,2-diacyl-3-β-D-galactosyl-*sn-*glycerol acylhydrolase (DGL – 3.1.1.26), triacylglycerol acylhydrolase (LIP - 3.1.1.3) and diacylglycerol O-acyltransferase (DGAT - 2.3.1.20).

**Fig. 8:**
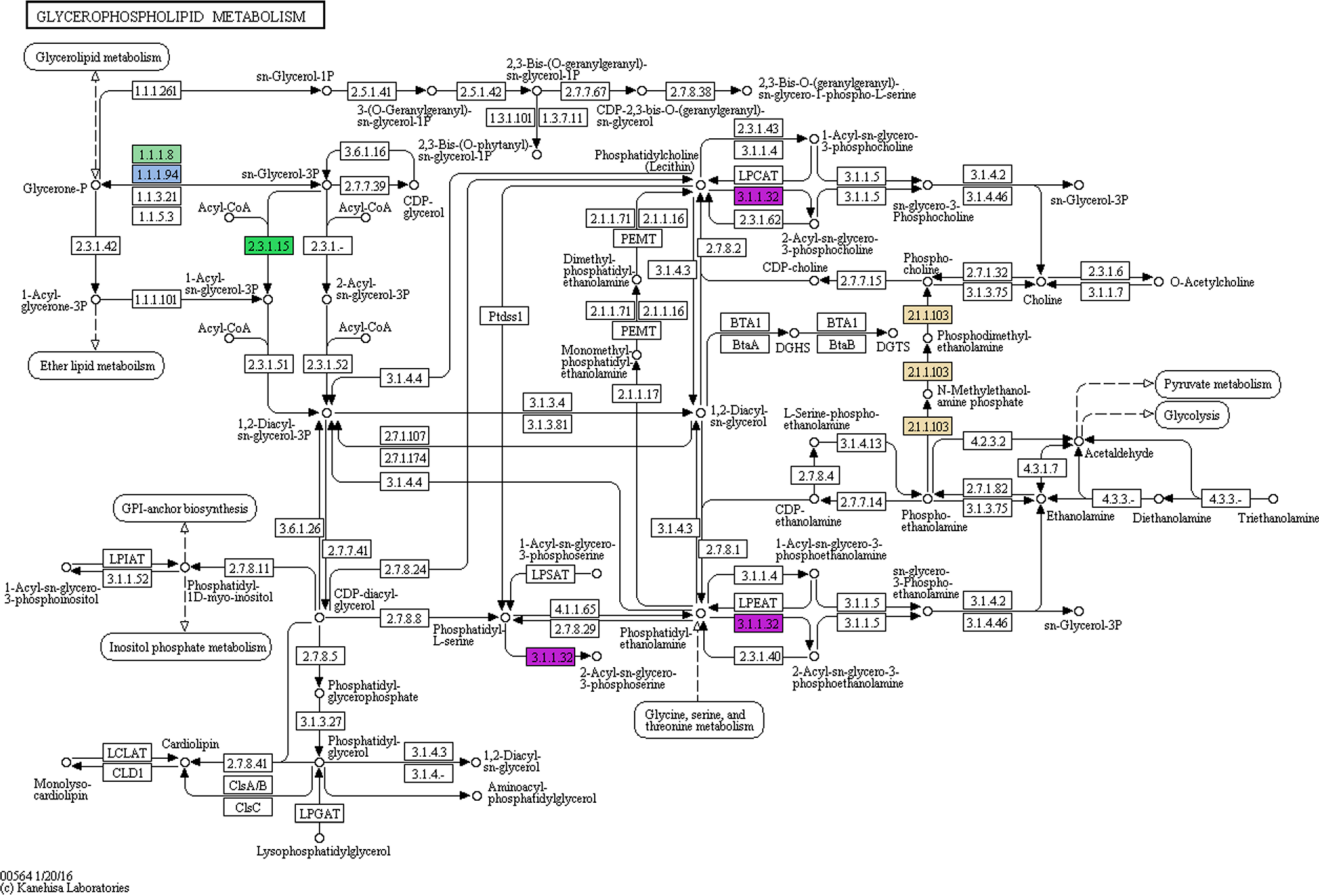
Enzymes target by QsMYB1 in the glycerophospholipid metabolism (colored boxes). Numbers correspond to enzymes E.C. numbers

Several genes encoding for enzymes related with the glycerophopspholipid metabolism (Fig. 8) were also identified: glycerol-3-phosphate dehydrogenase (NAD+) (GPDH - 1.1.1.8), glycerol-3-phosphate dehydrogenase [NAD(P)+] (GPDH - 1.1.1.94), phospholipase A1 (PLA1–3.1.1.32) and the phosphoethanolamine N-methyltransferase (NMT – 2.1.1.103).

### MYB1 target genes related with lipid transporters

Our ChIP-Seq data reveals that QsMYB1 is targeting several ATP-binding cassette proteins (ABC) or ABC-like transporters. Of these, 26 present high homology for the G family of ABC (ABCG) genes (Additional file 4). Five of these genes have high similarity with *AtABCG11*, six of them with *AtABCG37* and six with the *AtABCG40*. In addition, we identified five genes encoding for lipid-transfer proteins (LTPs) (Additional file 5), three of them with similarity to protease inhibitor/seed storage/LTPs and two of them putative LTPs genes in *Q. suber*.

### Relative expression of QsMYB1 targeted genes

**Fig. 9:**
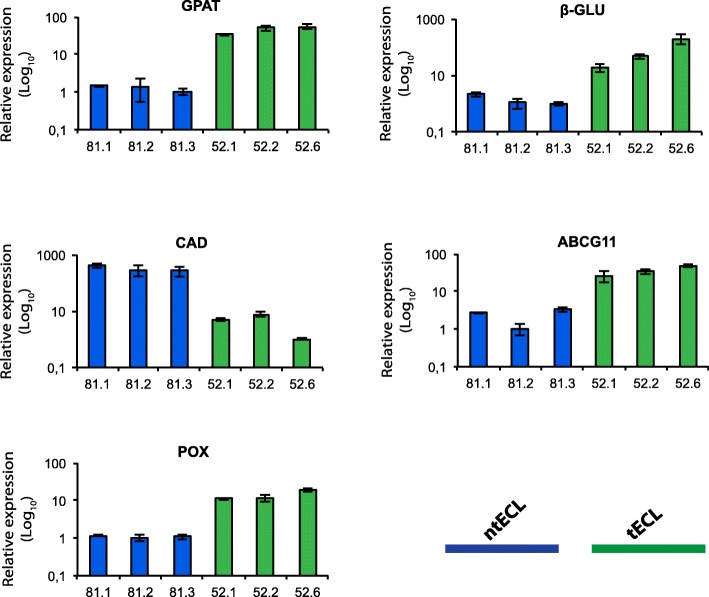
Relative transcripts levels five QsMYB gene targets quantified by RT-qPCR from transformed embryogenic cell lines (tECL) (52.1, 52.2 and 52.6) and non-transformed embryogenic cell lines (ntECL)(81.1–81.3). Mean and standard errors of three independent experiments are shown

The expression of five putative genes targeted by QsMYB1: *QsGPAT*, *Qsb-GLU*, *QsCAD*, *QsABCG11* and *QsPOX* were evaluated by RT-qPCR on transformed embryos overexpressing QsMYB1 (tECL) and non-transformed embryos (ntECL)(Fig. 9). Results shows relative increased expression when genes are targeted in 5’UTR (*QsGPAT*, *QsABCG11* and *POX*) and in the promotor region (*Qsβ-GLU*). In contrast, *QsCAD* which is targeted in an exonic region exhibited a relative decreased expression in embryos overexpressing QsMYB1::3xFLAG.

## Discussion

*Quercus suber* MYB1 (QsMYB1) is a TF that is mainly expressed in organs and tissues with secondary growth resulting from the activity of the phellogen [27]. This points to a putative regulation of QsMYB1 in the lignin and suberization processes and therefore possibly in cork formation and differentiation. In fact, *Arabidopsis* mutants generated by gene trap insertion in *AtMYB68,* the *QsMYB1* orthologue, produce increased biomass and lignin levels relatively to wild type. In addition its closest homolog, *AtMYB84,* exhibit an overlapping expression suggesting a partly redundant function [33].

In order to identify the genes regulated by QsMYB1 in a genome-wide scale, a cork oak embryo model was established, allowing protein overexpression, isolation and easy access to genomic material. In this context, genetic modified cork oak embryos overexpressing QsMYB1 were produced and used in a ChIP-Seq experiment allowing for the identification of the QsMYB1 putative binding sites. Although this is a proxy model, as embryos do not present neither the developmental stage nor the secondary growth tissues where *QsMYB1* is naturally up-regulated, it allowed for the identification of several QsMYB1 target genes with specific and defined DNA binding domains related with several aspects of tissue lignification, suberization and consequently with cork formation, which are further discussed. Furthermore QsMYB1 may not target all the binding sites as at the secondary tissues where it has been found to be expressed [27]. However, the overexpression system used in this study, which forces the production of QsMYB1, is an attempt to mimic the up-regulation and DNA-binding status observed in other tissues or organs [27]. The results showed that for five QsMYB1 gene targets analysed by RT-qPCR there is a pronounced gene expression modulation in the embryos overexpressing QsMYB1. These findings confirm that QsMYB1 regulates its expression. Moreover, it was observed that, for the tested genes, when QsMYB1 targets a gene region upstream of the coding sequence (e.g. 5’UTR and promoter) the gene expression is up-regulated whereas when the target region is an exon, the targeted gene expression is down-regulated (Fig. 9). Thus, QsMYB1 seems to have a dual opposite regulatory role either up-regulating or down-regulating gene expression.

### QsMYB1 targets phenylpropanoid pathway genes regulating the biosynthesis of essential lignin and suberin precursors

The results of our study clearly show that QsMYB1 is targeting genes coding for key enzymes responsible for the biosynthesis of phenylpropanoids, which constitute phenolic components of suberin and lignin polymers. Members of the R2R3-MYB TF family are well known for their regulatory function in diverse metabolisms as the phenylpropanoid [34], lignin [35, 36], lipid [37] and flavonoid metabolism [38] among others. Phenylpropanoids are phenolic derivatives which contain a phenyl ring and a C_3_ side chain that comprise a multitude of plant secondary metabolites and cell wall components as lignin and suberin [39, 40]. The *Arabidopsis MYB41,* phylogenetically close to *AtMYB68* (the Arabidopsis homolog of *QsMYB1*), when overexpressed has an up-regulatory effect on phenylpropanoid, lignin and suberin synthesis and related-genes expression [12]. Like AtMYB41, our results indicate an involvement of QsMYB1 in the phenylpropanoid metabolism. QsMYB1 targets 4-coumarate:CoA ligase which is involved in the conversion of ferrulic, caffeic and coumaric acids to ω-OH-alkyl-ferulates/n-alkyl ferulates, n-alkyl caffeates and n-alkyl coumarates, that in turn are linked to fatty alcohols to produce alkyl hydroxycinnamates, an essential constituent in suberin-associated waxes [41]. In addition, QsMYB1 targets cinnamyl alcohol dehydrogenase and class III plant peroxidase, which together with 4-coumarate:CoA are involved in the biosynthesis of the monomeric precursor of lignin *p*-hidroxy-cinnamyl alcohols, the so-called monolignols. Furthermore class III plant peroxidases are also involved in polymerization of suberin monomers in the apoplast [42]. A recent work demonstrated that various genes for enzymes of the phenylpropanoid pathway are up-regulated in the poplar phellem tissue, namely 4-coumarate:CoA ligase and cinnamyl alcohol dehydrogenase [13]. Together, the results indicate an involvement of QsMYB1 in the synthesis of phenolic compounds from the phenylpropanoid pathway evidencing an earlier regulatory action in the synthesis of essential lignin and suberin precursors.

### QsMYB1 is targeting genes encoding enzymes responsible for suberin biosynthesis

Suberin may be defined as a complex glycerol-based polymer consisting of a polyaliphatic polyester linked with phenolic components and embedded waxes [43–45]. Besides suberin composition vary between developmental stages, tissue and plant species, in *Q. suber* depolymerisation of cork suberin include monoacylglycerols of ω-hydroxy acids and α,ω-dicarboxilic acids, ferrulic acid linked to ω-hydroxi acids, trimeric diesters of glycerol linked to α,ω-dicarboxilic acids and ω-hydroxi acids linked to ferrulic acid and glycerol [46, 47]. Our results reveal that QsMYB1 is targeting genes that encode enzymes involved in synthesis of the suberin polyester building blocks; one *QsLACS* and two *QsKCS* predicted genes, constituting evidence of genes associated with suberin monomers synthesis in *Q. suber*. LACS catalyses the acyl activation reaction of free fatty acids to fatty thiosteres [48]. In poplar *LACS1* and *LACS2* are up-regulated in phellem [13]. In *Arabidopsis LACS1* and *LACS2* have overlapping roles activating fatty acids in cutin and wax synthetic pathways. In addition, *LACS2* is involved in suberin formation besides the capability of LACS enzymes may also act on modified fatty acids as ω-hydroxy acids and α,ω-dicarboxilic acids before esterification to glycerol [49]. In turn, β-ketoacyl-CoA synthase (KCS) is responsible for controlling the extension of elongation of the long-chain fatty acyl-CoAs [50]. In *Arabidopsis*, two β-ketoacyl-CoA synthases, *AtKCS2* and *AtKCS20* are involved in elongation of C_20_ acyl chain suberin precursors [51, 52]. In potato, silencing of *StKCS6* gene resulted in reduction of suberin monomers with chain lengths of C_28_ and higher in the tuber periderm indicating that StKCS6 is involved in elongation of suberin precursors to C_28_ or higher chain lengths [53].

Several CYP86 family members were identified in our data, namely homologues of *AtCYP86A1* and of *AtCYP86A8.* CYP86A8 monooxigenase are capable of ω-hydroxylating saturated and unsaturated fatty acids with chain lengths from C_12_ to C_18_ and is likely to be involved in cutin biosynthesis [54]. Two major monomers of the suberin polyester are ω-hydroxy acids and α,ω-dicarboxylic acids, which in plants are formed by the hydroxiliation of the terminal methyl group (ω-position) catalysed by enzymes of the CYP86 subfamily of cytochrome P450 monooxygenases [55]. The *Arabidopsis* CYP86A1 and CYP86B1 are involved in root suberin synthesis [55–57]. Similarly, in potato, the StCYP86A33 orthologue of AtCYP86A1 is important for the production of ω-functionalized suberin monomers of the tuber periderm resulting in significant reduction in 18:1 ω-hydroxy acids and α,ω-dicarboxilic acids when the gene is silenced [58].

Our results reveal a *QsGPAT* homolog target by QsMYB1 indicating a regulatory role of this TF in the production of monoacylglycerols, which may be considered as the initial step in the aliphatic suberin biosynthesis. In poplar, several *GPATs* are up-regulated in phellem, namely *GPAT5*, *GPAT6* and *GPAT8* [13]. In *Arabidopsis*, GPAT4, 6 and 8 are bifunctional enzymes with *sn*-2 acyltransferase and phosphatase activity, catalysing the formation of *sn*-2 monoacylglycerol products. GPAT5 in turn is involved in the transfer of very long-chain aliphatics to a glycerol based acceptor in *Arabidopsis* root and seed coat suberin [59]. *Arabidopsis* mutants of *GPAT5* show a large reduction in long-chain unsubstituted fatty acids, ω-hydroxy acids and α,ω-dicarboxilic acids in root and seed coat suberin with a total reduction of 50% in total suberin content [59]. In the case of GPAT7, the *GPAT7* gene is induced by wounding producing soluble suberin-like precursors in overexpressing lines [60]. These results clearly indicate that QsMYB1 is targeting genes encoding for enzymes that are responsible for the production of aliphatic domain of suberin.

### QsMYB1 targets putative transporters of suberin-lipid components

By directly targeting genes coding for 4-coumarate:CoA, cinnamyl alcohol dehydrogenase, LACSs, KCSs, GPATs amongst others, QsMYB1 is likely to regulate the phenylpropanoid and the suberin biosynthesis pathways. In addition, QsMYB1 may have a regulatory effect in the expression of suberin transporters.

The various suberin lipidic precursors are generically transported from the endoplasmic reticulum where they are synthesized to and across the plasma membrane, and then polymerized in the apoplast to form the suberin barrier [61]. We identified several members of the ABCG gene family targeted by QsMYB1, namely homologs of *AtABCG11* and *AtABCG6,* which implicates directly QsMYB1 in regulation of lipid transport and suberin formation. Transport of aliphatic lipids across the plasma membrane involves plasma membrane-localized transporters of the ABCG family which consists of half-transporters that oligomerise to form the functional transporter [62]. Rains et al. [13], recently reported up-regulated ABCG transporter genes in poplar phellem, namely homologs of *AtABCG11* and *AtABCG2*. In *Arabidopsis*, the ABCG transporter ABCG11 is implicated with cutin and suberin biosynthesis [63–65] while ABCG12 and ABCG13 are involved in export of cuticular wax monomers [65, 66]. Because homo- and heterodimers of ABCG11 and ABCG12 are reported as important for wax export and ABCG13 seems to be required for cutin deposition in flowers [67], it is widely accepted that tissue-dependent combinations of different half-size ABCG proteins involved in the transport of wax, cutin and suberin components are possible to occur [61]. Furthermore, ABCG11 can form dimers with ABCG9 and ABCG14 which links it to a function in lipid homeostasis regulation in vascular organs as phloem [68]. Additionally, a recent study reported that half-size ABCG2, ABCG6 and ABCG20 are involved in the formation of suberin layers in seed coats and roots where *abcg2/6/20* triple mutants had increased permeability, altered suberin structure and reduce aliphatic components [69]. Another type of proteins involved in lipid excretion into the extracellular space are LTPs. Evidence of LTPs involvement in cuticular wax, suberin and sporopollenin assembly is increasing in recent literature [70, 71]. Our data also identified five genes coding for LTPs reinforcing the involvement of QsMYB1 in the regulation of genes involved in lipid traffic across the cellular membrane and possibly in suberin assembly and deposition.

### Putative regulation of glycerolipids metabolism by QsMYB1

Glycerolipids are fatty acid esters of glycerol. Fatty acids are constituents of membrane lipids of every cell. The first step in *the novo* synthesis of fatty acids is the carboxylation of acetyl-CoA to malonyl-CoA by acetyl-CoA carboxylase [72]. In *Arabidopsis* acetyl-CoA carboxylase is required for very long chain fatty acid elongation [73]. Our data suggests that QsMYB1 is regulating a homolog gene of acetyl-CoA carboxylase, which indicates direct regulation on a fundamental key step of fatty acid synthesis and elongation pathways. The second enzyme responsible for fatty acids biosynthesis is the fatty acid synthase (FAS) which in plants is a type II FAS consisting of a multienzyme complex of acyl carrier proteins [72]. The results also revealed that at least 2 genes with homology for *Arabidopsis* enoyl-[acyl-carrier-protein] reductases (NADH) are QsMYB1 gene targets. One acyl-[acyl-carrier-protein] desaturase was also identified in our data. This type of desaturases are responsible for fatty acid modifications introducing *cis*-double ligation in the acyl chain acyl-[acyl-carrier-protein] and ultimately in the correspondent fatty acid [72]. Therefore, QsMYB1 may regulate the production of unsaturated fatty acids, which in turn is used as precursors for the suberin monomeric units synthesis.

Suberin, in cork oak, presents monoacylglycerols, diglycerol diesteres and diacylglycerols in is chemical composition [40, 46]. The availability of glycerol-derived molecules may constitute a critical limitation step in glycerol-derived monomers synthesis of suberin [59]. In this study, were identified two genes coding for aldehyde dehydrogenases which are responsible for the production of D-glyceraldehyde, which in turn is converted to glycerol by aldehyde reductase and alcohol dehydrogenase (both also identified in our data) to form glycerol in plants [72]. These results indicate that QsMYB1 is acting on transcription regulation of genes involved in the synthesis of glycerol. In addition, the results revealed that QsMYB1 is exerting its action on a gene encoding for diacylglycerol O-acyltransferase which is responsible for the conversion of diacylglycerols to triacylglycerols [74]. Interestingly, QsMYB1 also seems to act on the opposite conversion reaction of triacylglycerols to the correspondent diacylglycerols by targeting the homolog gene of a triacylglycerol acylhydrolase. Triacylglycerol acylhydrolases are also described to have the ability for catalysing the reaction of diacylglycerols to monoacylglycerols [75, 76]. This finding may constitute an evidence for the transcription regulation of diacylglycerols and monoacylglycerols synthesis in *Q. suber*, however further studies have to be performed in order to confirm this hypothesis.

### QsMyb1 may be involved in abiotic stress responses through the regulation of the glycerophospholid pathway

Glycerophospholipids are major components of cellular membranes which are synthesized from glycerol-3-phosphate and have functions as second messengers in plant growth regulation and cellular response to environmental change or stress [72]. Lipid membrane composition, remodelling and activation of a variety of phospholipid-based pathways is a strategy developed by plants to survive and adapt to osmotic stress [77].

Almeida et al. [29] reported a putative function of QsMYB1 in the regulatory network of cork oak response to heat and drought stresses. Accordingly, we found that QsMYB1 is targeting a gene coding for phospholipase A1, a *sn*-1-specific phospholipase that releases free fatty acids from phospholipids and can act on the cellular membrane galactolipids and triacylglycerols [78]. In *Arabidopsis,* the *DEFECTIVE IN ATHER DEHISCENCE1 (DAD1)* gene has been shown to code phospholipase A1 catalysing the initial step of jasmonic acid biosynthesis [79]. The *Arabidopsis* phospholipase coded by the At2g42690 gene, which is UV-B inducible, was also reported as having properties that suggest a possible involvement in the biosynthesis of jasmonic acid [80]. These studies point to involvement of phospholipases A in hormonal response to stress and may indicate evidence of a relation between QsMYB1 and response to stress mediated by hormones. Furthermore, our results revealed that QsMYB1 is targeting a *phosphoethanolamine N-methyltransferase* homolog gene of *Arabidopsis* which catalyses key steps in choline biosynthesis, namely the N-methylation of phosphoethanolamine [81]. Choline is well reported as an important player in plant grow and development [82]. Choline is also oxidized to glycinebetaine which is a strong osmoprotectant that confers tolerance to salinity, drought and other stress [83, 84]. In *Arabidopsis*, the silencing *of phosphoethanolamine N-methyltransferase* results in temperature-sensitive male sterility and salt hypersensitivity, which demonstrates that choline biosynthesis also contributes to tolerance to environmental stress [85]. Our results suggest an involvement of QsMYB1 response to drought stress specifically involving a key choline biosynthesis enzyme supporting the hypothesis that QsMYB1 expression is modulated in response to heat and drought stresses [29].

### QsMYB1 targets other transcriptional elements expanding its regulatory action

Three motifs ([CWHCAA], [CYTCBTC] and [BKTGG] were identified as the principal motifs characteristic of the QsMYB1-DNA binding. The [CWHCAA] and [BKTGG] motifs present high homology with already described R2R3-MYB binding site motifs [31], while [CYTCBTC] has a similarity with zinc finger C2H2-type motif of TF IIIA, which in *Arabidopsis* encodes a protein required for transcription of 5S ribosomal RNA [86]. However, the presence of secondary motifs signatures is a constant in the dataset. Secondary motifs result from two events: two TFs binding to neighbouring sites, or one TF binding to another TF that in turn binds to DNA. Taking into account that members of the MYB family are well known by acting as homo- or heterodimers [87], the data suggest that QsMYB1 is either binding to DNA or to other TFs resulting in an extensive and complex transcriptional regulation of genes. Therefore, QsMYB1 may be involved in a complex regulatory network targeting both transcriptional elements and a group of specific genes with direct function in several biological processes. We found that QsMYB1 is targeting other transcription elements, which supports the idea that QsMYB1 directly regulates the expression of genes and also exerts its effect through other TFs, transcription regulators and chromatin regulators. Amongst these, the more abundant gene targets of QsMYB1 are involved: in fine tuning transcription, translation and posttranscriptional modifications as CCHC zinger finger proteins (CCHC) family, which specifically interacts with single-stranded DNA or RNA oligonucleotides [88] or C2H2-type zinc fingers TFs (C2H2) which besides DNA binding also provides protein-protein and RNA-protein interactions [89] or BED-type zinc finger proteins, which function as either transcription activators or repressors by modifying local chromatin structure on binding to GC-rich sequences [90, 91]; in regulation of cell division, cell-fate determination, transmembranar signaling and vesicle fusion as the WD40-like TFs; and in integrating gene regulatory networks which controls the metabolic, hormonal and environmental signals in plant growth, development and response to plant abiotic and biotic stresses as the AP2/ERF TF [92, 93] and the bHLH TF families [87].

Together with MYB, bHLH represents a large proportion of the TFs in *Arabidopsis* both regulating multiple biological process [94]. Members of the two families are described by acting as homodimers or heterodimers consisting of proteins from the same family or in complexes with TFs from other families including the WD40-like TFs previously referred [94]. In many cases, MYB/bHLH complexes have been described and associated with the developmental and metabolic plasticity present in plants [94]. The regulation of pathways controlled by these two types of TFs as well as the mechanism regulating their activity is therefore linked, which is in line with our results. Thus, by forming heterodimers with other transcriptional elements, QsMYB1 may trigger additional regulatory mechanisms at chromatin, transcription and post-transcriptional level.

## Conclusions

This study has contributed to increase the knowledge about the molecular mechanisms behind cork formation and differentiation. To address the role of QsMYB1 we performed a genome-wide analysis of the DNA targets of this TF using ChIP-Seq. QsMYB1 was shown to be involved in lignin and suberin biosynthesis and assembly, acting through a complex regulatory network directly by targeting enzyme genes, and indirectly by targeting genes encoding for other transcriptional regulatory elements. The complex QsMYB1 regulatory mechanisms that we disclosed may represent an example of how *Q. suber* has optimised its developmental regulatory systems during evolution. Furthermore, we found QsMYB1 as a master regulatory factor of cork formation and differentiation since it acts on genes belonging to the lignin and suberin biosynthetic pathways, the two main components of cork. In addition, QsMYB1 has the capability to modulate the process of lignin and suberin synthesis through the regulation of specific genes of the phenylpropanoid pathway. Further studies are required to confirm and reveal the specific role of the reported genes and to clarify the exact function of QsMYB1 in the process. Even so, the generated data is a starting point to an in-depth understanding of suberin biosynthesis and deposition. By starting to unravel the biosynthetic pathways involved in cork formation we provide important knowledge to support the development of breeding programs based e.g., on the design of cork metabolic engineering strategies.

## Methods

### Plant material and growth conditions

No specific permission was needed for the development of this study. *Quercus suber* is protected in Portugal against logging. Sample collections did not involve endangered or other protected species.

A stable *Q. suber* somatic embryogenesis line, Ce1, was obtained by somatic embryogenesis induction from adult tree leaves as described by Hernandéz et al. [95].

Trees were located at Cercal do Alentejo, Portugal, growing in a *montado* ecosystem. Embryo clusters were grown under long day conditions (16 h light per 8 h dark cycle) at 25 °C on MSSH medium, containing Murashige and Skoog (MS) micronutrients [96], Schenk and Hildebrandt (SH) macronutrients [97] and supplemented with MS vitamins [96], 3% (*w*/*v*) sucrose and 1% (w/v) agar.

### *Quercus suber* genetic modified cell lines

A binary vector for overexpression of QsMYB1::triple FLAG epitope fusion protein was constructed using Gateway™ technology (Gateway® Recombination Cloning, Invitrogen, Lifetechnologies Corporation, CA, USA). QsMYB1 coding sequence (accession number JF970262) was amplified with primers MYB1ns_attB1F and MYB1ns_attB2R (Additional file 6: Table S1) using attB1 and attB2 adapters and recombined with the pDONR221 vector to generate a QsMYB1 ENTRY vector (pENTRY_QsMYB1). Triple FLAG epitope (3xFLAG) nucleotide sequence was generated by annealing the 3xFLAG_F_Olig and the 3xFLAG_R_Olig oligonucleotides (Additional file 6: Table S1) and recombined with the pDONR221 vector in order to produce a 3xFLAG ENTRY vector (pENTRY_3xFLAG). A PCR-fusion/Gateway procedure for gene fusion [98] was applied to generate an overexpression pK7WG2,0 vector carrying the QsMYB1::3xFLAG fused coding sequence (pK7MYB1::3xFLAG). A diagram of the procedure is showed in (Additional file 6: Figure S1). The resulting binary vector was mobilized into *A. tumefaciens* AGL1 by triparental mating [99] using *E. coli* MC1012 harbouring the mobilizing plasmid pRK2013. After a 10 days sub-culture period, embryos were transformed as described by Álvarez and Ordás (2007) [100].

### RT-qPCR validation of QsMYB1::3xFLAG expression

Genetic transformed embryo clusters were grown by repetitive embryogenesis in kanamycin selective medium during 24 months with regular sub-culturing every 30 days, to allow for transformed embryos selection. After 24 months of embryo transformation, aleatory independent embryo clusters were selected for RT-qPCR analysis in order to screen the expression of the QsMYB1::3xFLAG transcript. Specific primers qPCRQsMYB1F and SR(C) (Additional file 6: Table S2) were used in the RT-qPCR experiments. One microgram of total RNA was used for cDNA synthesis using the QuantiTect Reverse Transcription kit (Qiagen, Valencia, CA, USA), which includes an additional genomic DNA elimination step and uses a mix of oligo(dT) and random hexamer primers. RT-qPCR experiments were carried out in a iCycler iQ5 Instrument (Bio-Rad Laboratories, Hercules, CA, USA) using the Sso Advanced Universal SYBR Green Master mix (Bio-Rad Laboratories, Hercules, CA, USA) in 96-well plates. Three replicates were performed in reaction mixtures of 20 μL containing 10 μL of 2X Sso Advanced Universal SYBR Green Master mix, 400 nM of each specific primer pair (Forward/Reverse) and 1 μL of cDNA with a dilution of 1:100 as template. Normalization between samples was performed using two reference genes: ACTIN (ACT) and CLATHRIN ADAPTOR COMPLEXES (CACs) shown to be constitutively expressed (data not shown). Normalized relative quantities (NRQ) were calculated by $\mathrm{NRQ}=\frac{{\mathrm{E}}_{\mathrm{goi}}^{\Delta  \mathrm{Ct},\mathrm{goi}}}{\sqrt[\mathrm{f}]{\prod_{\mathrm{o}}^{\mathrm{f}}{\mathrm{E}}_{{\mathrm{ref}}_{\mathrm{o}}}^{\Delta  \mathrm{Ct},\kern0.5em {\mathrm{ref}}_{\mathrm{o}}}}}$ where E is the amplification efficiency for each primer pair, f the number of reference genes used to normalize the data, goi the gene of interest, ref the reference gene and ∆Ct is the Ct of the sample with higher Ct across samples minus the Ct value of the sample in test [101].

### RT-qPCR of QsMYB1 target genes

Three random transformed embryo clusters overexpressing QsMYB1::3xFLAG and three random non-transformed embryo clusters were selected for assessment of the relative expression of 5 QsMYB1 putative target genes: *QsGPAT, Qsβ-GLU*, *QsCAD*, *QsABCG11* and *QsPOX* genes. Specific primers for each gene (Additional file 7) were used in the RT-qPCR experiments. RNA and cDNA were prepared as for RT-qPCR validation of QsMYB1::3xFLAG expression. RT-qPCR experiment were performed by the same described conditions, using as reference genes ELONGATION FACTOR 1α (EF1-α) and β-TUBILIN (β-TUB). Normalized relative quantities (NRQ) were calculated as described in the previous section.

### Detection of QsMYB1::3xFLAG fused protein in *Q. suber* embryos

Expression of full-length QsMYB1 protein fused with 3xFLAG tag was confirmed by Western Blot. Total protein content of genetic modified and non-genetic modified embryos was extracted using TCA-acetone method [102]. Supernatants were collected, and total protein concentrations quantified according to Lowry method [103], using BSA as protein standard. Protein components of cell lysates (25–40 μg protein) were separated on sodium dodecyl sulfate 10% polyacrylamide gel, and then transferred onto polyvinylidene difluoride (PVDF) membranes (Amersham Biosciences, Buckinghamshire, UK). PVDF membranes were blocked with 5% (*w*/*v*) of non-fat dry milk at room temperature for 1 h, and incubated overnight at 4 °C with a mouse monoclonal ANTI-FLAG® M2 (Sigma-Aldrich, St Louis, MO) primary antibody (1:2000). Bands were visualized by chemiluminescence using anti-mouse horseradish peroxidase-conjugated secondary antibodies, and developed with ECL reagents (Amersham Biosciences, Buckinghamshire, UK), according to manufacturer’s instructions.

### Immunodetection

*Quercus suber* embryos were fixed in a freshly prepared solution of 4% paraformaldehyde in PBS (phosphate buffered saline: 137 mM NaCl; 0.27 mM KCl; 1 mM phosphate buffer, pH 7.4). The fixed embryos were sectioned (10- to 20-μm-thick) under water using a vibratome. The sections were kept at 4 °C in a 0.1% solution of formaldehyde in PBS until further use. Subsequently, sections were incubated with blocking solution (5% (w/v) Bovine Serum Albumin (BSA) in 1× PBST – PBS supplemented with 0.5% (*v*/v) Tween 20), and after washing in 1xPBST were incubated with a mouse monoclonal ANTI-FLAG® M2 (Sigma-Aldrich, St Louis, MO) primary antibody (1:200) supplemented with 1% BSA for 16 h at 4 °C. The primary antibody was detected with a goat polyclonal secondary antibody to mouse - Alexa Fluor® 488 IgG - H&L (ab150113, Abcam Cambridge, UK) (1:200 dilution in PBST supplemented with 1% BSA) for 1 h at 37 °C. Sections were mounted with Vectashield mounting medium with DAPI (Vector Laboratories, USA). Images were acquired on an epifluorescence microscope Axio Imager.Z1.

### ChIP-Seq assay

ChIP-Seq assay of *Q. suber* embryos expressing QsMYB1::3xFLAG was performed by cross-linking and isolating chromatin embryos using the Abcam Plant Chromatin Extraction Kit (ab156906, Abcam, Cambridge, UK) protocol, with slight modifications to the cross-linking procedure: starting with 3 g of fresh embryos, 1 volume of Plant RNA Isolation Aid (Thermo Fisher Scientific, Waltham, MA, USA) per unit mass of embryo tissue (mL/mg) was added to 1% formaldehyde solution and vacuum infiltration step was performed during 20 min at 4 °C. Chromatin was sheared with a Bioruptor® Plus (Diagenode): high intensity pulses, 5 rounds of 10 cycles, 30 s ON / 30 s OFF, with a sample volume of 150 μL to generate ~ 300-bp average fragment sizes, as determined by agarose gel and capillary electrophoresis (Agilent 2100 Bioanalyzer) using the High Sensitivity DNA ChIP kit (Agilent, 5067–4626). ChIP was performed followed by the protocol developed by Li and collaborators [104] using mouse monoclonal ANTI-FLAG® M2 antibody (Sigma-Aldrich, St Louis, MO) and mouse IgG serum (Sigma-Aldrich, St Louis, MO), generating ChIP and Mock samples, respectively. Mock samples were generated in the same experimental conditions, except the immunoprecipitation step that was performed with IgG serum instead of the ANTI-FLAG® M2 specific antibody. After reverse cross-link and DNA purification, the precipitated DNA was quantified and analysed by capillary electrophoresis (Agilent 2100 Bioanalyzer) using the High Sensitivity DNA ChIP kit (Agilent, 5067–4626).

### Library preparation and Illumina sequencing

DNA from ChIP of 3xFLAG (ChIP) and from ChIP of IgG serum (Mock) was used to produce sequencing libraries using the KAPA Hyper Prep Kit (KAPA Biosystems, Wilmington, MA). A total of four single-end libraries were prepared, one for each of the two replicates of the ChIP experiment and two of the control (Mock), and sequenced using the HiSeq 4000 system (Illumina, San Diego, CA), in two independent sequencing lanes (L1 and L5), with a read length of 50 bp.

### Sequencing data pre-processing and read mapping

FastQC [105] was used as the first data control tool to check the quality of the sequencing reads. Raw reads were preprocessed with Sickle [106] using 20 for the quality threshold and 40 for the read length. Mapping of the reads against a draft version of the cork oak genome (Additional file 8) was performed with Bowtie2 [107] and unique mapped reads were extracted using the mapping quality value of 255. Library complexity was measured by the non-redundant fraction (NRF) and PCR Bottlenecking Coefficients (PBC). NRF was calculated as described by Nakato and Shirahige [108] by setting the threshold for non-redundant reads to 2. Non-redundant reads were extracted using the output of MACS2 [109] filterdup utility. PBC metrics were obtained using BEDtools [110] genomecov. Phantompeakqualtools [4] was applied for the cross-correlation analysis of the ChIP and Mock libraries.

### Peak calling and downstream processing

An irreproducible discovery rate (IDR) analysis was employed to find the most consistent set of peaks across replicates. Self-pseudoreplicates and pooled-pseudoreplicates were also generated and peaks were called with Model Based Analysis for ChIP-Seq data (MACS2) [109], using a q-value < 0.05, based on a Poisson distribution comparing the ChIP and Mock sequenced samples. A maximum number of 8 duplicates tags were defined, as it seemed the appropriate value for which the number of peaks reached a plateau phase using the fragment length estimated by Phantompeakqualtools (Additional file 9). IDR v2.0.2 [111] was used to measure the reproducibility of replicates ranking the peaks by their *p*-value with an IDR threshold of 0.05. Peaks passing the IDR threshold by comparing true replicates were selected for downstream analysis according to the ENCODE (Encyclopedia of DNA elements) ChIP-Seq guidelines [4].

Based on the structural annotation generated for the genome draft (Additional file 8), custom python scripts were created to assign a gene to each IDR peak and its respective location regarding gene boundaries. Promotor regions were defined to be up to 2000 bp upstream of the beginning of each gene and terminator regions were set to be up to 1000 bp downstream of the end of each gene.

### Analysis of targets related with transcriptional elements

Identification of target genes encoding for TFs, transcription regulators and chromatin regulators were analyzed with the PlantTFcat tool [32] submitting the gene sequences correspondent of each peak to the web platform analysis.

### Motif analysis

A motif discovery analysis was performed using MEME-ChIP software [112]. MEME-ChIP analysis was done with the gene models available for the cork oak draft genome (Additional file 8) and selected based on the location of detected peaks. Sequences with 250 bp from both sides of peaks summits were retrieved and these 501 bp sequences were given as input in MEME-ChIP software to identify common motifs. Once motifs were identified, the motifs comparison tool TOMTOM [113] was used to compare with already identified and annotated motifs as the Arabidopsis motifs detected in protein microarray (PBM) [31] or Arabidopsis motifs identified by DNA affinity purification sequencing (DAP-Seq) [30].

### Metabolic pathway analysis

Genomic sequences with 500 bp from both sides of peak summits were checked against the NCBI non-redundant protein (Nr) database, annotated with gene ontology plant-terms, enriched and refined using ANNEX function and used for metabolic pathway analysis using through the Blast2GO software [114]. Metabolic pathway analysis was then performed using the Blast2GO interface to access Kyoto Encyclopedia of Genes and Genomes (KEGG) database [115]. Pathway maps were downloaded and inspected manually focusing in the identified enzymes of pathways related with suberin and lignin metabolism, namely phenylpropanoid biosynthesis, fatty acid biosynthesis and degradation, glycerolipid metabolism and glycerophospholipid metabolism.

## Additional files

Additional file 1: Fragmented DNA. DNA fragments after chromatin fragmentation visualized and quantified by agarose gel (A) and Bioanalyzer (B). (PDF 2085 kb)

Additional file 2: Types of transcriptional regulators. Families and number of transcriptional regulators targeted by QsMYB1. (XLSX 52 kb)

Additional file 3: Enzyme peaks. Detected peaks and annotation related with enzyme coding genes. (XLSX 15 kb)

Additional file 4: ABCG peaks. Detected peaks and annotation related with ABCG genes. (XLSX 11 kb)

Additional file 5: LTPs peaks. Detected peaks and annotation related with LTPs genes. (XLSX 40 kb)

Additional file 6: Gene construction strategy and primers. **Figure S1.** Gene construction strategy used for QsMYB::triple FLAG epitope fusion protein production. **Table S1.** Primers used to generate the overexpression destination vector pK7MYB1::3xFLAG. **Table S2.** Primers used to confirm the integration of the foreign DNA delivered by the destination vector plasmid and to quantify the QsMYB1::3xFLAG transcript by RT-qPCR. (DOCX 101 kb)

Additional file 7: List of primers used in RT-qPCR experiments of *QsGPAT*, *Qsβ-GLU*, *QsCAD*, *QsABCG11* and *QsPOX* genes. (DOCX 12 kb)

Additional file 8: Cork oak genome. Description of cork oak genome draft generation. (DOCX 26 kb)

Additional file 9: IDR analysis. Description of Irreproducible discovery rate (IDR) analysis. (DOCX 40 kb)

## Abbreviations

AP2/ERF: APETALA2/−Ethylene Responsive element binding factor
BED-type (Zn): BED-type zinc finger proteins
bHLH: Basic helix-loop-helix
bZIP: Basic leucine zipper
C2H2: C2H2-type zinc fingers TFs
CCHC: CCHC zinc finger proteins
ChIP: Chromatin immunoprecipitation
ChIP-Seq: Chromatin immunoprecipitation followed by high-throughput DNA sequencing
FAS: Fatty acid synthase
LTPs: Lipid-transfer proteins
MYB: Myeloblastosis
RT-qPCR: Quantitative real time PCR
TF(s): Transcription factor(s)
UTR: Untranslated regions
