# Supplementary material for: ChIP-Seq reveals that QsMYB1 directly targets genes involved in lignin and suberin biosynthesis pathways in cork oak (Quercus suber)
Source: BMC Plant Biol. 2018 Sep 17;18:198. doi: 10.1186/s12870-018-1403-5 (PMC6142680; doi:10.1186/s12870-018-1403-5)
Supplement: Supplementary file 1 — Fragmented DNA. DNA fragments after chromatin fragmentation visualized and quantified by agarose gel (A) and Bioanalyzer (B). (PDF 2085 kb) [file 12870_2018_1403_MOESM1_ESM.pdf]

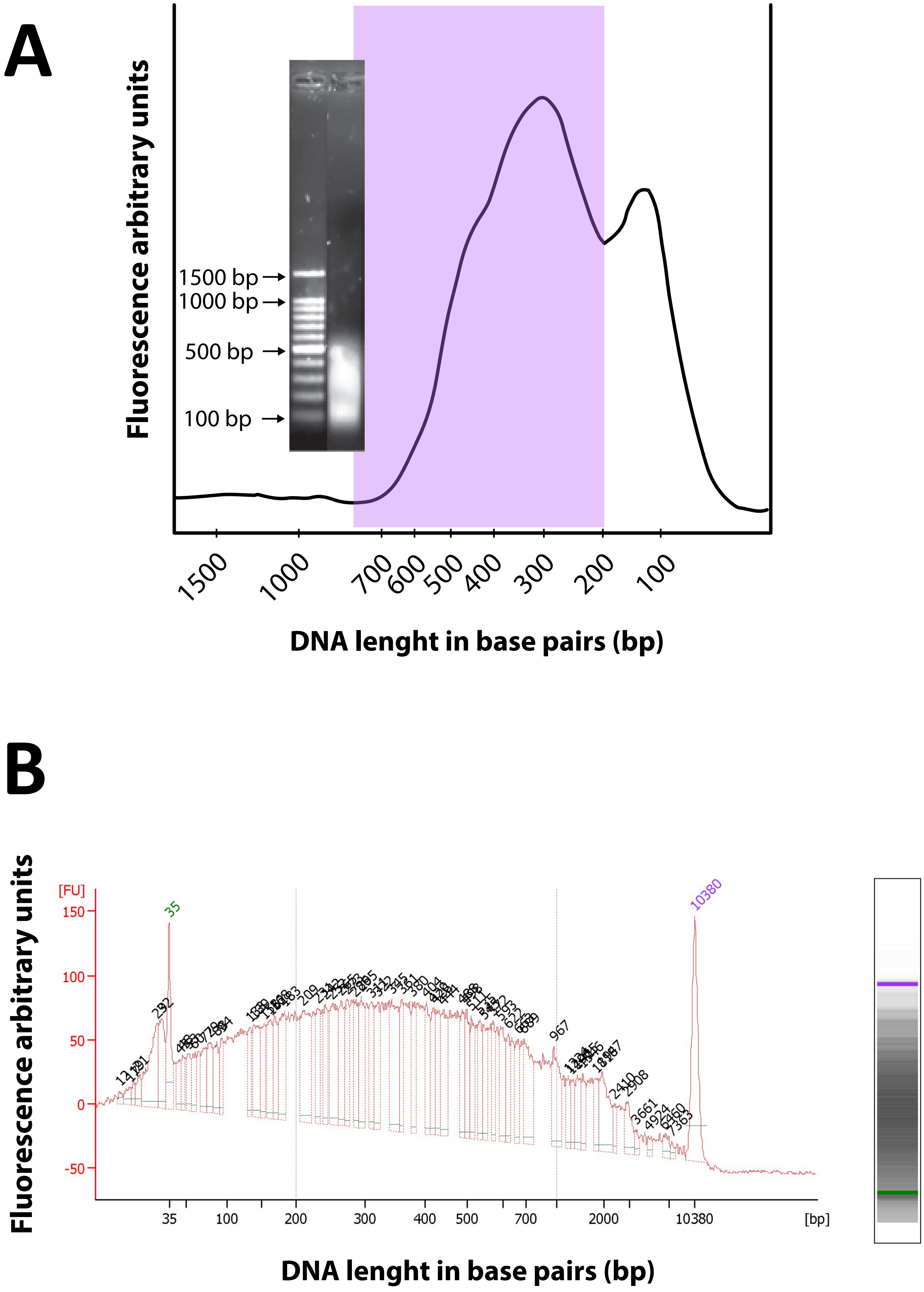
A
Fluorescence arbitrary units
1500 bp
1000 bp
500 bp
100 bp
1500
1000
700
600
500
400
300
200
100
DNA lenght in base pairs (bp)
B
Fluorescence arbitrary units
[FU]
150
100
50
0
-50
35
10380
967
35
100
200
300
400
500
700
2000
10380
[bp]
DNA lenght in base pairs (bp)
