# Supplementary material for: ChIP-Seq reveals that QsMYB1 directly targets genes involved in lignin and suberin biosynthesis pathways in cork oak (Quercus suber)
Source: BMC Plant Biol. 2018 Sep 17;18:198. doi: 10.1186/s12870-018-1403-5 (PMC6142680; doi:10.1186/s12870-018-1403-5)
Supplement: Supplementary file 6 — Gene construction strategy and primers. Figure S1. Gene construction strategy used for QsMYB::triple FLAG epitope fusion protein production. Table S1. Primers used to generate the overexpression destination vector pK7MYB1::3xFLAG. Table S2. Primers used to confirm the integration of the foreign DNA delivered by the destination vector plasmid and to quantify the QsMYB1::3xFLAG transcript by RT-qPCR. (DOCX 101 kb) [file 12870_2018_1403_MOESM6_ESM.docx]

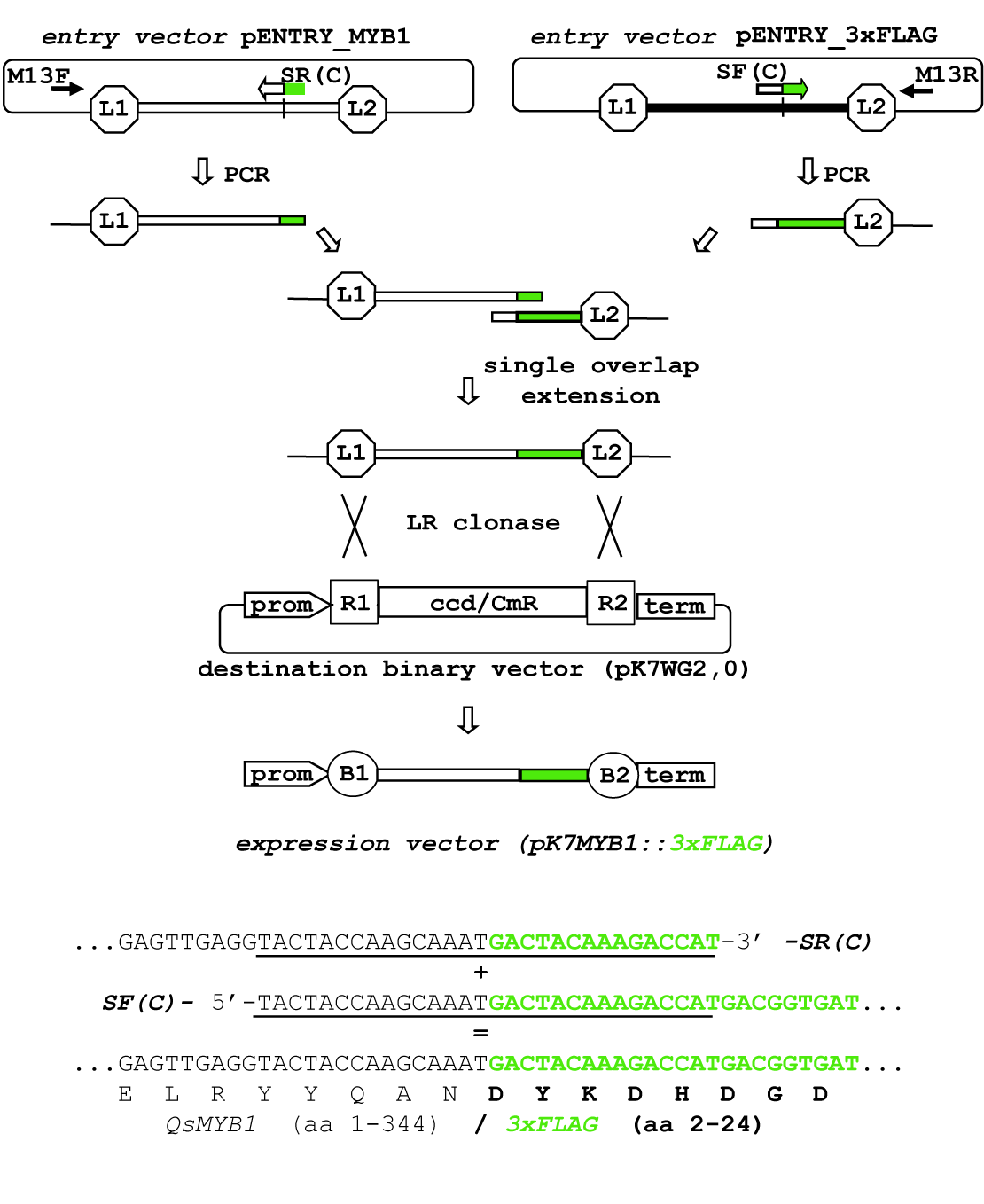


Figure S1 – Gene construction strategy used for QsMYB::triple FLAG epitope fusion protein production.

Table S1 - Primers used to generate the overexpression destination vector pK7MYB1::3xFLAG

| **Primer** | **Sequence** |
| --- | --- |
| M13F | 5’-gtaaaacgacggccagt-3’ |
| M13R | 5’-CAG GAA ACA GCT ATG AC-3’ |
| SF(C) | 5’-TAC TAC CAA GCA AAT GAC TAC AAA GAC CAT-3’ |
| SR(C) | 5’-ATG GTC TTT GTA GTC ATT TGC TTG GTA GTA-3’ |
| 3xFLAG_F_Olig | 5’- AGCTTAGACTACAAAGACCATGACGGTGATTATAAAGATCATGACATCGATTACAAGGATGACGATGACAAGTGATATCG -3’ |
| 3xFLAG_R_Olig | 5’- GATCCGATATCACTTGTCATCGTCATCCTTGTAATCGATGTCATGATCTTTATAATCACCGTCATGGTCTTTGTAGTCTA -3’ |
| MYB1ns_attB1F | 5’- AAAAAGCAGGCTTAGAAGGAGATAGAACCATGGGGAGAGCTCCATGTTGTGACAAAG -3’ |
| MYB1ns_attB2R | 5′- AGAAAGCTGGGTCATTTGCTTGGTAGTACCTCAACTCT -3’ |
| attB1 adapter | 5′- GGGGACAAGTTTGTACAAAAAAGCAGGCT -3′ |
| attB2 adapter | 5’- GGGGACCACTTTGTACAAGAAAGCTGGGT -3′ |

Table Ss2 - Primers used to confirm the integration of the foreign DNA delivered by the destination vector plasmid and to quantify the QsMYB1::3xFLAG transcript by RT-qPCR

| **Primer** | **Sequence** |
| --- | --- |
| qPCRQsMYB1F | 5’-AGCCTAAAGCAAGAGATGAAGAGAG-3’ |
| SR(C) | 5’-ATG GTC TTT GTA GTC ATT TGC TTG GTA GTA-3’ |
| nptIIF | 5’-GAGGCTATTCGGCTATGACTG-3’ |
| nptIIR | 5’-ATCGGGAGCGGCGATACCGTA-3’ |
| virGF | 5’-AAGGTGAGCCGTTGAAACAC-3’ |
| virGR | 5’-ATCTCAAGCCCATCTTCACG-3’ |
