# Supplementary material for: ChIP-Seq reveals that QsMYB1 directly targets genes involved in lignin and suberin biosynthesis pathways in cork oak (Quercus suber)
Source: BMC Plant Biol. 2018 Sep 17;18:198. doi: 10.1186/s12870-018-1403-5 (PMC6142680; doi:10.1186/s12870-018-1403-5)
Supplement: Supplementary file 7 — List of primers used in RT-qPCR experiments of QsGPAT, Qsβ-GLU, QsCAD, QsABCG11 and QsPOX genes. (DOCX 12 kb) [file 12870_2018_1403_MOESM7_ESM.docx]

List of primers used in RT-qPCR experiments of QsGPAT, Qsβ-GLU, QsCAD, QsABCG11 and QsPOX genes.

| **Primer Name** | **Primer Sequence (5’ – > 3’)** | **Gene RefSeq accession** |
| --- | --- | --- |
| GPAT_Fwd | ATATCCTCACACAGAAGAC | XM_024044471.1 |
| GPAT_Rev | TACCAGAGTGTTCTACTAGC |  |
| β-GLU_Fwd | GTTCTAGTTGCACAGTTG | XM_024058024.1 |
| β-GLU_Rev | CTTTCTGTACTTCCTGATAC |  |
| CAD_Fwd | GAGATTCACTCCACTAACTA | XM_024062723.1 |
| CAD_Rev | TATACACCCAACTCCTACTA |  |
| ABCG11_Fwd | AGCTAACACAGAGTTTGTAG | XM_024021169.1 |
| ABCG11_Rev | ATTACTGAGACTGACCATC |  |
| POX_Fwd | GTTAGGATTGTTGAGGTC | XM_024039011.1 |
| POX_Rev | CTTATACACACTCAGCTTCT |  |
