# Supplementary material for: ChIP-Seq reveals that QsMYB1 directly targets genes involved in lignin and suberin biosynthesis pathways in cork oak (Quercus suber)
Source: BMC Plant Biol. 2018 Sep 17;18:198. doi: 10.1186/s12870-018-1403-5 (PMC6142680; doi:10.1186/s12870-018-1403-5)
Supplement: Supplementary file 8 — Cork oak genome. Description of cork oak genome draft generation. (DOCX 26 kb) [file 12870_2018_1403_MOESM8_ESM.docx]

**Generation of cork oak genome draft**

**DNA extraction and sequencing**

Nuclear DNA extractions were performed from leaf material using the innuPREP Plant DNA Kit ([Analytik Jena](https://www.analytik-jena.de/)). A total of nine paired-end (PE) libraries and nine mate-pair (MP) libraries, of different insert sizes, were prepared and sequenced using the Illumina Hiseq2000 platform and a read length of 100 bp (PE) or 50 bp (MP), which yielded a total of 4,451,211,066 reads (Table S3).

**Preprocessing and evaluation of high-throughput sequence data**

Sickle [1] was used to filter out the reads, using as criteria minimum quality (20) and read length (80 bp and 40 bp for the PE and MP libraries, respectively). Furthermore, any read with an ambiguous base (N) was also discarded (Table 1). After removal of the low quality reads a total of 83.1% (PE) and 85.3% (MP) from the initial number of reads remained in the dataset. Genome size size estimations were done with the PE reads using SGA preqc module [2], which extends the k-mer count based analysis by including methods to explicitly distinguish true genomic k-mers from artificial k-mers containing sequencing errors. The predicted genome size was 715.9 Mb, which represented a smaller size when compared with previous flow cytometry estimations [3].

**Genome assembly and scaffolding**

Paired-end reads were assembled with Ray v2.3.1 [4] with a k-mer size of 81. Mate-pair libraries were mapped onto the Ray assembly with bowtie2 v2.2.7 [5] and those alignments were used to scaffold the genome using BESST v1.3 [6]. The cork oak genome draft assembly contained 50,595 scaffolds, with a minimum size of 1 kb. The total genome length contained in the draft assembly was 655.5 Mb, and the percentage of Ns was 5.6%. The assembly N50 was 87,392 bp, while the longest scaffold contained 665,812 bp.

**Assembly validation and annotation**

The 50,592 scaffolds were used in a CEGMA analysis, which showed that the majority of core genes were already present in the draft (81.9% in full length and 96.4% partially represented). Augustus was used for gene prediction using *Arabidopsis thaliana* as the training model. A total of 109,847 genes were detected, of which 90,978 (82.8%) represented full length features (with a start and stop codon) and 18,869 (17.2%) were partial. Rapsearch2 annotations against NCBI-nr and NCBI plants and an Interproscan run displayed 73,630 (67.0%), 70,792 (64.5%) and 71,913 (65.5%) genes with a match, respectively.

**Table S3**

| **Sample Name** | **Number of raw reads** | **Insert size (bp)** | **Cleaned reads** | **% Kept** |
| --- | --- | --- | --- | --- |
| WHABPI001738-44 | 352,588,096 | 170 | 315,869,872 | 89.59% |
| HL8.2 | 321,172,452 | 170 | 290,834,920 | 90.55% |
| HL8.4 | 309,545,950 | 170 | 280,005,244 | 90.46% |
| WHAIPI001737-45 | 212,784,794 | 500 | 169,685,260 | 79.75% |
| HL8.5 | 334,949,460 | 500 | 276,589,646 | 82.58% |
| HL8.6 | 376,329,674 | 500 | 332,800,750 | 88.43% |
| HL8.7 | 218,435,846 | 800 | 160,517,058 | 73.48% |
| HL8.8 | 228,831,248 | 800 | 165,292,408 | 72.23% |
| HL8.9 | 175,494,582 | 800 | 128,782,240 | 73.38% |
| Total paired-end | 2,530,132,102 | - | 2,120,377,398 | 83.81% |
| HL8.10 | 217,902,622 | 2,000 | 189,416,746 | 86.93% |
| HL8.14 | 228,392,402 | 2,000 | 196,420,348 | 86.00% |
| HL8.16 | 224,235,198 | 2,000 | 195,654,282 | 87.25% |
| Sob19 | 277,442,886 | 2,000 | 236,518,096 | 85.25% |
| Sob20 | 189,991,228 | 2,000 | 161,768,988 | 85.15% |
| Sob21 | 281,147,166 | 2,000 | 237,842,630 | 84.60% |
| Sob15 | 197,695,978 | 5,000 | 168,796,986 | 85.38% |
| Sob17 | 158,274,708 | 5,000 | 129,562,092 | 81.86% |
| Sob18 | 145,996,776 | 5,000 | 122,714,074 | 84.05% |
| Total mate-pair | 1,921,078,964 | - | 1,638,694,242 | 85.30% |

**Bibiography**

1. Joshi NA FJ. Sickle: A sliding-window, adaptive, quality-based trimming tool for FastQ files [Internet]. 2011. Available: https://github.com/najoshi/sickle

2. Simpson JT. Exploring Genome Characteristics and Sequence Quality Without a Reference. 2013; Available: http://arxiv.org/abs/1307.8026

3. Zoldos V, Papes D, Brown SC, Panaud O, Siljak-Yakovlev S. Genome size and base composition of seven *Quercus* species: inter- and intra-population variation. Genome. NRC Research Press Ottawa, Canada ; 1998;41: 162–168. doi:10.1139/g98-006

4. Boisvert S, Laviolette F, Corbeil J. Ray: simultaneous assembly of reads from a mix of high-throughput sequencing technologies. J Comput Biol. Mary Ann Liebert, Inc.; 2010;17: 1519–33. doi:10.1089/cmb.2009.0238

5. Langmead B, Salzberg SL. Fast gapped-read alignment with Bowtie 2. Nat Methods. Nature Publishing Group, a division of Macmillan Publishers Limited. All Rights Reserved.; 2012;9: 357–9. doi:10.1038/nmeth.1923

6. Sahlin K, Vezzi F, Nystedt B, Lundeberg J, Arvestad L. BESST--efficient scaffolding of large fragmented assemblies. BMC Bioinformatics. 2014;15: 281. doi:10.1186/1471-2105-15-281
