# Supplementary material for: ChIP-Seq reveals that QsMYB1 directly targets genes involved in lignin and suberin biosynthesis pathways in cork oak (Quercus suber)
Source: BMC Plant Biol. 2018 Sep 17;18:198. doi: 10.1186/s12870-018-1403-5 (PMC6142680; doi:10.1186/s12870-018-1403-5)

**Irreproducible discovery rate (IDR) analysis**

As IDR requires relaxed thresholds for initial peak calling, we defined a maximum number of duplicate tags in MACS2 to 8, as it seemed the appropriate value for which the number of peaks reached a plateau (Figure 1).

**Figure 1:** Variation on the number of peaks detected by MACS2 by changing the number of duplicate reads permitted.


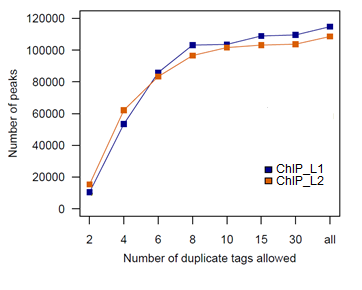

Supplement: Supplementary file 9 — IDR analysis. Description of Irreproducible discovery rate (IDR) analysis. (DOCX 40 kb) [file 12870_2018_1403_MOESM9_ESM.docx]
